# Comparison of clinical outcomes between sorafenib and hepatic artery infusion chemotherapy in advanced hepatocellular carcinoma: A STROBE-compliant article

**DOI:** 10.1097/MD.0000000000010611

**Published:** 2018-04-27

**Authors:** Min Kyu Kang, Jung Gil Park, Heon Ju Lee

**Affiliations:** Division of Gastroenterology and Hepatology, Department of Internal Medicine, Yeungnam University College of Medicine, Daegu, Korea.

**Keywords:** chemotherapy, hepatocellular carcinoma, progression-free survival, sorafenib

## Abstract

Sorafenib is the most widely used multikinase inhibitor in patients with advanced hepatocellular carcinoma (HCC). Despite its efficacy, only a small proportion of patients experience tumor regression. Hepatic artery infusion chemotherapy (HAIC) can be used as an alternative treatment for HCC.

A total of 139 patients with advanced HCC, treated with HAIC (HAIC group, n = 95) or sorafenib (sorafenib group, n = 44), were retrospectively analyzed in a single hospital. We compared the efficacy and overall survival (OS) between the 2 groups, and investigated the factors affecting response rate in the HAIC group.

The objective response rate (ORR) was significantly higher in the HAIC group than in the sorafenib group (23.2% vs 2.3%; *P* *=* .01). The progression-free survival time was longer in the HAIC group than in the sorafenib group (274 vs 166 days; *P* *=* .03). However, there was no significant difference in OS between the 2 groups (359 vs 223 days; *P* *=* .05). In the multivariate analysis, international normalized ratio (INR), serum bilirubin, and presence of objective response were significant prognostic factors associated with OS (*P* *=* .03, *P* *=* .01, and *P* *=* .01, respectively). In the HAIC group, INR, nonobjective response group, and < 4 HAIC cycles were identified as independent risk factors of OS (*P* *=* .03, *P* *=* .01, and *P* *=* .01, respectively).

The ORR in patients treated with HAIC was found to be superior to that in advanced HCC patients treated with sorafenib. Better tumor response and prolonged OS can be expected in patients who receive ≥ 4 HAIC cycles.

## Abstract 2

Supplemental Digital Content is available in the text

## 1 Introduction

Hepatocellular carcinoma (HCC) has been reported to have the sixth highest cancer prevalence and the third highest cancer-related mortality worldwide.^[1]^ Prognosis in patients with very early and early-stage HCC is favorable, because they can usually be treated with curative treatment, including liver transplantation, surgical resection, and local ablation therapy.^[2,3]^ However, unfortunately, most patients with HCC are diagnosed at the intermediated or advanced stage, at which the prognosis is poor.^[2]^ Moreover, patients with advanced HCC have a median survival time of less than 12 months. The prognosis of untreated patients is very poor, with a median survival time of less than 6 months.^[4,5]^

Sorafenib, which is an oral multikinase inhibitor, has been shown to have efficacy of increasing the overall survival (OS) as a systemic treatment in patients with advanced HCC classified as Child–Pugh A or B, performance status 1 or 2, portal vein thrombosis (PVT), and lymph node and distant metastasis by the Barcelona Clinic Liver Cancer (BCLC) staging system.^[6–8]^ Despite the efficacy of sorafenib in advanced HCC, alternative treatments are required due to the unsatisfactory survival rate and tumor response in the Asia-Pacific region.^[5,9]^ In Asian countries, hepatic artery infusion chemotherapy (HAIC) with an implantable port system has been used as an alternative treatment in limited patients with advanced HCC.^[10]^ In patients treated with HAIC, the chemotherapeutic agent can reach the HCC directly through the port system, as the HCC receives most of its blood supply through the hepatic artery.^[10]^ Theoretically, it is possible to accumulate a high concentration of chemotherapeutic agent in the liver, while its systemic concentration is lower than that of systemic intravenous chemotherapy.^[9,10]^ In previous studies, HAIC showed a 20% to 30% treatment response rate and better survival rate compared with sorafenib monotherapy in patients with advanced HCC without distant metastasis.^[11–13]^ However, there are currently no randomized controlled studies comparing the clinical efficacy and survival benefit between HAIC and sorafenib.^[5,11–14]^

For these reasons, in the present study, we compared the tumor response, OS, progression-free survival (PFS), and safety between advanced HCC patients treated with HAIC and sorafenib.

## 2 Methods

### 2.1 Patients

A total of 355 patients with advanced HCC treated with HAIC (n = 227) and sorafenib (n = 128) at Yeungnam University Hospital from January 2000 to December 2016 were included, with following criteria: age 20 to 80 years; PVT, lymph node, and distant metastasis; an Eastern Cooperative Oncology Group performance status 1 or 2; preserved liver function below Child–Pugh grade B; and intermediate-stage HCC, which is not eligible for transcatheter arterial chemoembolization.

Of these, 216 patients were excluded, based on the following criteria: other malignant tumors except HCC; serious medical condition such as cardiopulmonary or renal insufficiency; previous systemic intravenous chemotherapy; and <2 cycles of HAIC. A total of 95 patients treated with HAIC and 44 treated with sorafenib were analyzed in this study. HCC was diagnosed by a liver biopsy or typical radiologic features in computed tomography (CT) or magnetic resonance imaging (MRI) according to the guideline in American Association for the Study of Liver Diseases.^[2]^ This study was approved by the institutional ethics committee of Yeungnam University.

### 2.2 Treatment protocol

#### 2.2.1 HAIC group

Chemotherapeutic agents were repeatedly administrated into the arterial catheter inserted through the right femoral artery by a trained radiologist. Cisplatin (JW Pharmaceutical, Seoul, Korea, 25 mg/m^2^ for 12 hours on days 1–4) and 5-fluorouracil (5-FU, JW Pharmaceutical, Seoul, Korea, 750 mg/m^2^ for 12 hours on days 1–4) were delivered into the port system every 4 weeks. Intravenous hydration was performed before the infusion of chemotherapy to prevent cisplatin-induced nephrotoxicity with antiemetic treatment with 5-hydroxytryptamine3 receptor antagonists in all patients. The dosages of chemotherapeutic agents were changed depending on the severity of adverse events. The following cycle of treatment was reduced by 25% in case of grade 2 toxicity and 50% in case of grade 3 toxicity. The treatment was stopped when the patient could not tolerate it, severe adverse events occurred, or the cancer progressed.

#### 2.2.2 Sorafenib group

Sorafenib (Nexavar; Bayer HealthCare, Leverkusen, Germany) was administrated orally at 400 mg twice a day. The patients were assessed for adverse events at our outpatient clinic every 1 or 2 months. The following cycle of treatment was reduced by 50% in case of grade 3 toxicity and the treatment was stopped for the same reasons as described for the HAIC group above.

#### 2.2.3 Study assessments

The tumor response was assessed by 3-phase contrast-enhanced CT or MRI after every 2 cycles in the HAIC group, and every 1 or 2 months in the sorafenib group. The treatment response was evaluated using the modified Response Evaluation Criteria in Solid Tumor (mRECIST) criteria.^[15]^ The treatment response was defined as follows: complete response (CR), the disappearance of any arterial enhancement of tumor; partial response (PR), at least a 30% decreased in the sum of diameters of viable portions; progressive disease (PD), an increase of 20% in the sum of diameters of viable portions; and stable disease (SD), any cases that did not qualify for either PR or PD.^[15]^ An objective response was defined as CR or PR, and disease control was defined as an objective response or SD. The treatment response was defined as the most effective response during the treatment period.

The primary endpoints were the OS and the PFS period for both groups. The OS was assessed as the time from the first treatment to death or final follow-up visit, and the PFS was assessed as the time from first treatment to radiological progression. The secondary endpoints were the objective response rate (ORR) and the disease control rate (DCR) between the 2 groups. To evaluate factors associated with OS and tumor response, we also analyzed the clinical characteristics of the HAIC group. The treatment-related toxicity was assessed by Common Terminology Criteria for Adverse Events 4.0.

### 2.3 Statistical analysis

All continuous variables are expressed as the mean with standard deviation or number of percentage. The categorical and continuous valuables were calculated using the Chi-square test, Fisher extract test, and Student test. Kaplan–Meier survival curves with a log rank test were performed for the analysis of OS and PFS in both groups. Univariate and multivariate analyses were performed to evaluate the factors associated OS and PFS using a Cox regression model, and the factors associated objective response in HAIC group were analyzed using a logistic regression model. Receiver operator characteristics curve analysis was performed to evaluate the optimal cycles of HAIC. A *P* value less than .05 was considered statistically significant. All statistical analyses were performed using R version 3.2.2 (R Foundation for Statistical Computing, Vienna, Austria).

## 3 Results

### 3.1 Baseline characteristics

Table 1 summarizes the baseline characteristics of the HAIC and sorafenib groups. Overall, the mean age was 55.7 ± 8.0 years and 87.1% of the patients were male. The most common etiology of the HCC was chronic hepatitis B virus infection (72.7%). Eighty-nine patients (64.0%) were classified as Child–Pugh class B and 50 patients (36.0%) were Child–Pugh class A. The Model for End-Stage Liver Disease score was 7.6 ± 3.7. The most common BCLC stage of the HCC was type C (69.8%). The largest tumor diameter was 7.9 ± 3.6 cm and 100 patients (71.9%) were found to have PVT. Distant metastasis occurred in 22.1% and 38.6% of patients in the HAIC and sorafenib groups, respectively. There were no statistically differences in any baseline characteristics between the 2 groups.

**Table 1:**
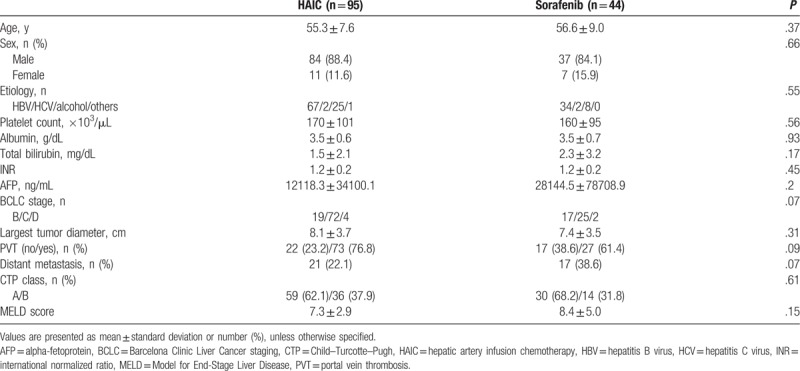
Baseline characteristics.

### 3.2 Differences in the treatment response between the HAIC and sorafenib groups

Table 2 summarizes the treatment response of the 2 groups. The number of patients who achieved CR, PR, SD, and PD were 2 (2.1%), 20 (21.1%), 32 (33.7%), and 41 (43.2%) in the HAIC group, respectively, and 0 (0.0%), 1 (2.3%), 16 (36.4%), and 27 (61.4%) in the sorafenib group, respectively. The ORR was significantly higher in the HAIC group than in the sorafenib group (23.2% vs 2.3%, *P* = .01). The DCR tended to be higher in the HAIC group than in the sorafenib group, but was not significantly different (56.9% vs 38.6%, *P* = .07).

**Table 2:**
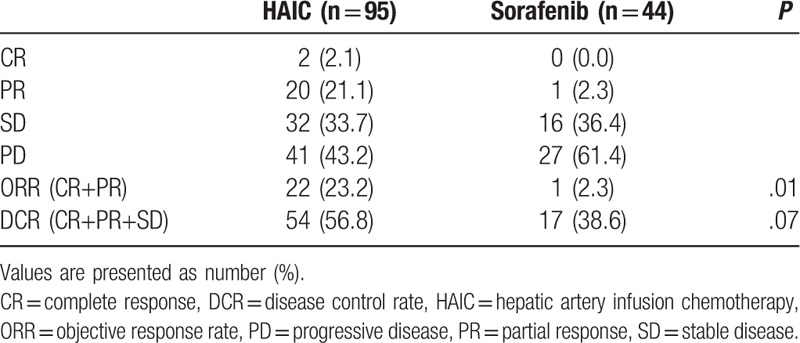
Treatment response in patients treated with HAIC or sorafenib.

### 3.3 Differences in the overall survival and progression-free survival rates between the HAIC and sorafenib groups

**Figure 1:**
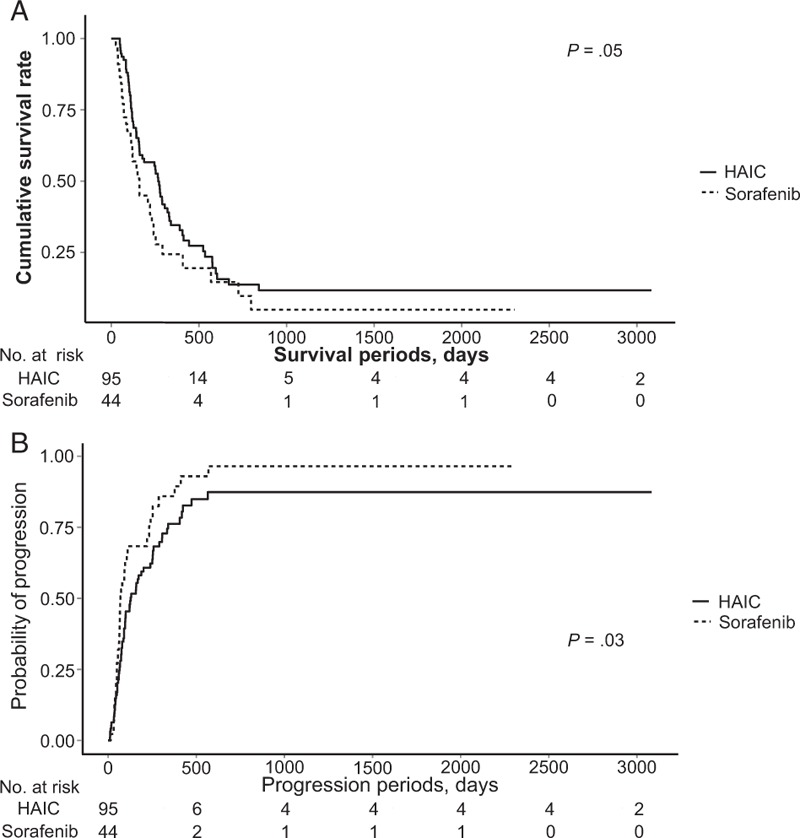
(A) Overall survival rates in patients treated with HAIC and sorafenib. (B) Progression-free survival rates in patients treated with HAIC and sorafenib. HAIC = hepatic artery infusion chemotherapy.

Figure 1 shows the differences in OS and PFS between the 2 groups. The median OS periods in the HAIC and sorafenib groups were 359 days [95% confidence interval (95% CI), 241–477 days] and 223 days (95% CI, 113–333 days), respectively. The cumulative OS rate tended to be better in the HAIC group than in the sorafenib group, but it was not significantly different (*P* = .05). The median PFS period in both groups was 274 days (95% CI, 156–392 days) and 167 days (95% CI, 60–273 days), respectively. The cumulative PFS rate was significantly better in the HAIC group than in the sorafenib group (*P* = .03).

In the univariate analysis, a better OS rate was associated with high serum albumin, low serum bilirubin, low international normalized ratio (INR), absence of PVT, and presence of an objective response. In the multivariate analysis, better OS rate was associated with low serum bilirubin, low INR, and presence of objective response (*P* = .01, *P* *=* .03, and *P* *=* .01, respectively, Table 3).

**Table 3:**
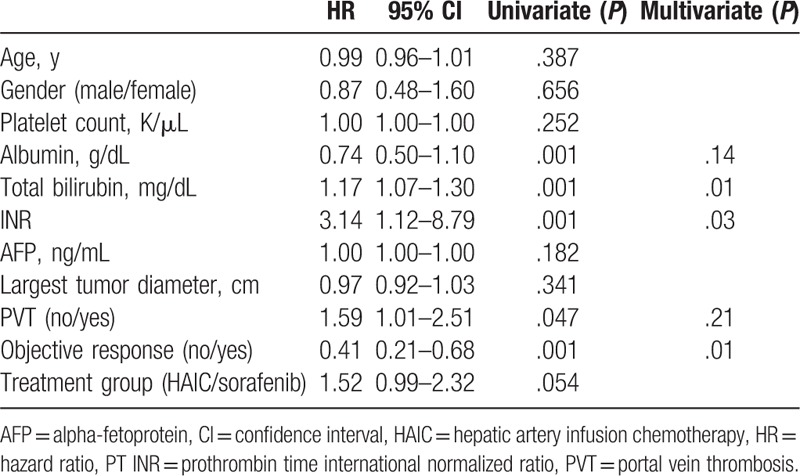
Factors associated with overall survival in patients treated with HAIC or sorafenib.

### 3.4 Overall survival rate, progression-free survival rates according to presence of objective response in the HAIC group

**Figure 2:**
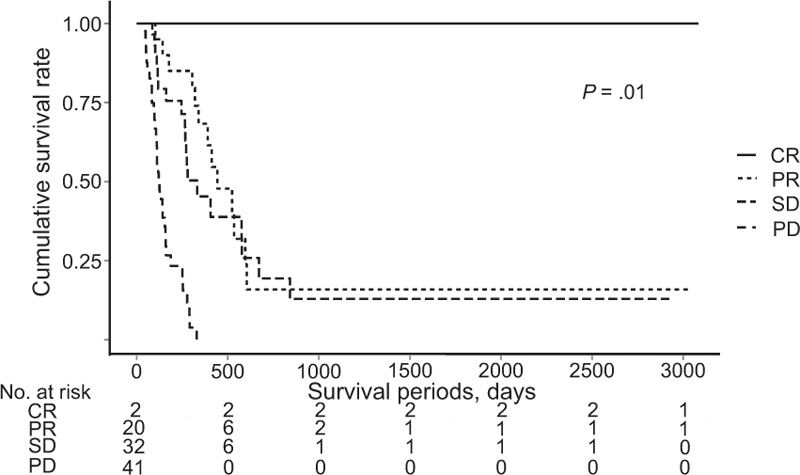
Overall survival rate according to treatment response in patients treated with HAIC. CR = complete response, HAIC = hepatic artery infusion chemotherapy, PD = progressive disease, PR = partial response, SD = stable disease.

The presence of an objective response was the only significant factor associated with both the OS and PFS rates. Moreover, among the 44 patients treated with sorafenib, only 1 patient achieved PR. Therefore, we evaluated the OS rate according to the treatment response in patients treated with HAIC (Fig. 2). The median OS periods in patients who achieved CR, PR, SD, and PD were 2872, 545, 373, and 135 days, respectively. The patients who achieved better treatment response had significantly better OS in the HAIC group (*P* = .01). Therefore, we compared the OS and PFS rates by dividing the patients treated with HAIC into 2 subgroups (objective response group vs nonobjective response group).

**Figure 3:**
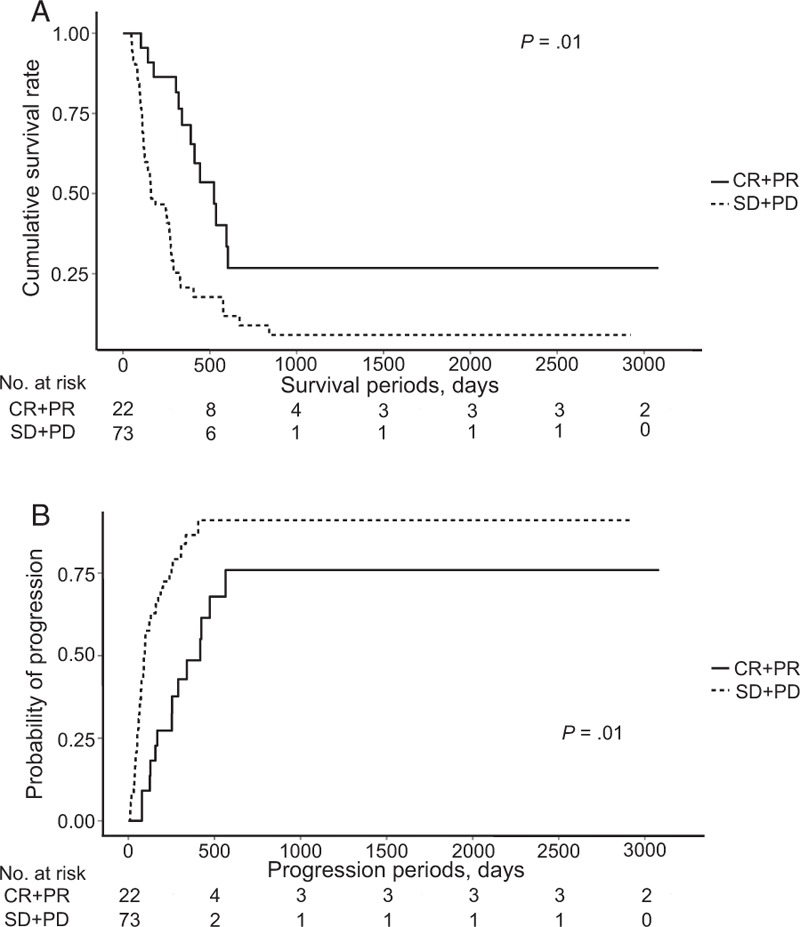
(A) Overall survival according to the presence or absence of an objective response in patients treated with HAIC. (B) Progression-free survival according to the presence and absence of objective response in patients treated with HAIC. CR = complete response, HAIC = hepatic artery infusion chemotherapy, PD = progressive disease, PR = partial response, SD = stable disease.

In the subgroup analysis in the HAIC group, cumulative the OS rate was significant better in the objective response group than in the nonobjective response group (*P* = .01, Fig. 3A). The median OS periods in the objective response group and nonobjective response group were 740 days (95% CI, 329–1151) and 239 days (95% CI, 155–324), respectively. The cumulative PFS rate is also significant better in objective response group than in nonobjective response group (Fig. 3B, *P* = .01). The median PFS period in both subgroups was 270 days (95% CI, 207–332) and 106 days (95% CI, 84–128), respectively. In the multivariate analysis, low INR, presence of an objective response, and ≥4 HAIC cycles were independent factors associated with better OS rate (*P* *=* .03, *P* = .01, and *P* = .01, respectively) (Table 4). In addition, HAIC cycle base on 4 times was the only independent prognostic factor associated with an objective response in the multivariate analysis (*P* = .01) (Supplementary table 1).

**Table 4:**
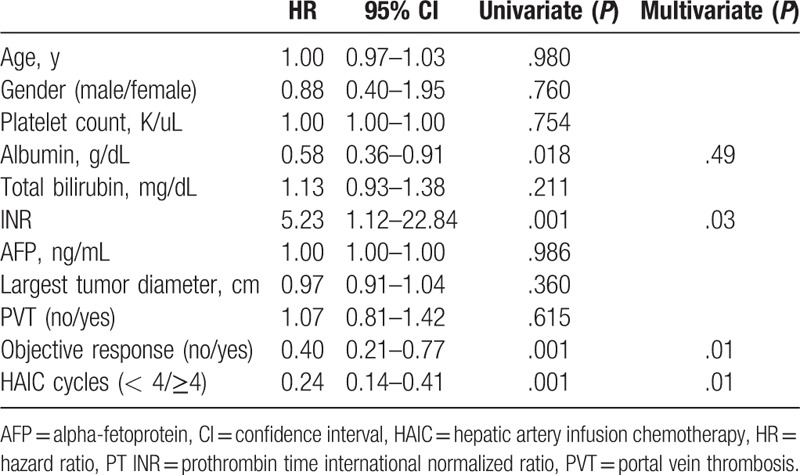
Factors associated with overall survival in patients treated with HAIC.

### 3.5 Significant adverse events between the HAIC and sorafenib groups

During the study period, a dose reduction was required in 46.3% and 54.5% in the HAIC group and sorafenib group, respectively (*P* = .4, supplementary table 2). The most common cause for dose reduction was fatigue in both groups (14.7% in the HAIC group and 11.4% in the sorafenib group). In the HAIC group, hematologic disorders (10.6%), including neutropenia, pancytopenia, and thrombocytopenia, were second most common cause for dose reduction, followed by hepatic failure (9.5%). In the sorafenib group, hepatic failure (9.4%) is the second common cause for dose reduction, followed by mucosal toxicity (5.8%) including hand foot syndrome, oral mucositis. The rate of discontinuation of the treatment was higher in the HAIC group than in the sorafenib group (32.6% vs 27.3%, *P* = .01, supplementary table 2).

## 4 Discussion

Sorafenib has been shown to have efficacy for improving the OS in patients with advanced HCC. However, only a limited number of patients achieved a partial response, with modest survival benefits, in 2 large phase III randomized clinical trials.^[6,7]^ Although HAIC tended to show a better response compared with sorafenib therapy in some small retrospective studies, there are currently no prospective studies that directly compare sorafenib with HAIC.^[5,11–14]^ In previous studies, the ORR ranged from 19% to 30.9% in patients treated with HAIC and from 2% to 3.3% in patients treated with sorafenib.^[5–7,11,13]^ In our study, the ORR was significantly higher in the HAIC group than in the sorafenib group (23.2% vs 2.3%, *P* = .01), which is consistent with the results of previous studies. Especially, unlike in the HAIC group, where no patient achieved a CR, the survival periods of the 2 patients (2.1%) who achieved a CR in the HAIC group were over 8 years. In previous studies, the DCR ranged from 38.1% to 71.3% in patients treated with HAIC and from 35.3% to 43% in the patients treated with sorafenib.^[5–7,11,13]^ However, in our study, although the HAIC group tended to achieve higher DCR than the sorafenib group, it did not reach statistical significance (56.9% vs 38.6%, *P* *=* .07). The mechanism of sorafenib involves targeting the pathways associated with progression and angiogenesis of the tumor, which seems to induce SD, but not PR or CR. The outcomes of our study, with only 1 patient achieving a PR, and with no case of CR, also support a limited ORR of sorafenib. Therefore, patients treated with HAIC are expected to achieve a better response than those treated with sorafenib.

Different results of OS rate and PFS rates are reported in various previous studies comparing HAIC and sorafenib in patients with advanced HCC.^[5,11,12,14]^ The use of different treatment protocols of HAIC and radiologic response assessments, including mRECIST or RECIST, might have caused the different outcomes in these studies. In studies conducted in Korea and Japan, the median OS in patients treated with HAIC and sorafenib ranged from 7.1 to 14 and from 6.5 to 10.7 months, respectively.^[5–7,11,12]^ Of these, a recent Japanese study reported a better OS rate in patients treated with HAIC than in those treated with sorafenib in patients with macrovascular invasion (17 vs 7 months).^[11]^ In addition, a Korean study reported a significantly longer median OS in patients treated with HAIC than those with sorafenib (7.1 vs 5.5 months).^[10]^ In our study, the median OS tended to be longer in the HAIC group than in the sorafenib group, although statistical significance was not reached (359 vs 223 days). However, the median PFS in the HAIC group was significantly longer than in the sorafenib group (274 vs 167 days). In 2 previous studies, the median PFS in the HAIC group was approximately 3 months, which is similar to the results of our study.^[5,12]^

In our study, presence of objective response, better baseline liver function indicating low serum bilirubin, and low INR was associated with better OS rate. However, the treatment modality did not affect the OS rate in the multivariate analysis. These results suggest that preserved liver function and presence of an objective response are more important than the choice of treating with HAIC or sorafenib for improving OS. Although HAIC did not show efficacy for improving the OS rate, the objective tumor response was an important factor associated with improved OS. In the subgroup analysis, preserved liver function, including low INR, presence of an objective response, and more than 3 HAIC cycles, was associated with a better OS rate in the HAIC group.

To continue HAIC treatment, adverse events are an important issue in patients with advanced HCC. Almost half of the patients needed to reduce the drug dosage in both groups. Fatigue was the most common cause of dose reduction in both groups. Characteristically, hematologic disorders, including neutropenia and thrombocytopenia, were more frequent in the HAIC group, whereas mucosal toxicity, including hand-foot syndrome and mucositis, was the main cause of dose reduction in the sorafenib group.

In our study, several limitations were noted. First, radiologic imaging including CT was performed relatively regularly, whereas blood chemistry results and information on the occurrence of adverse events were not regularly recorded. We therefore evaluated adverse events based on tests performed or recorded at the time of dose reduction. Second, few patients treated with sorafenib were relatively small compared with other studies. Third, this study was conducted in a single institution retrospectively. However, to our best knowledge, this study included the largest number of patients undergoing HAIC in a single institution.

In conclusion, HAIC treatment induces better tumor response compared with sorafenib in patients with advanced HCC. To achieve a better response rate, more than 3 HAIC cycles are needed. HAIC might be considered as an alternative treatment of sorafenib in selected patients with advanced HCC.

## Author contributions

H. J. L. and J. G. P. are guarantors of integrity of the entire study. H. J. L. and J. G. P. designed the study. M. K. K. collected and analyzed data. M. K. K. drafted the manuscript, which was critically revised by J. G. P.

**Conceptualization:** Heon Ju Lee.

**Formal analysis:** Min Kyu Kang.

**Funding acquisition:** Jung Gil Park.

**Investigation:** Jung Gil Park.

**Methodology:** Jung Gil Park.

**Project administration:** Jung Gil Park, Heon Ju Lee.

**Resources:** Min Kyu Kang.

**Software:** Min Kyu Kang.

**Supervision:** Jung Gil Park, Heon Ju Lee.

**Validation:** Min Kyu Kang, Heon Ju Lee.

**Visualization:** Min Kyu Kang.

**Writing – original draft:** Min Kyu Kang.

**Writing – review & editing:** Jung Gil Park.

## Supplementary Material

Supplemental Digital Content
