# Supplementary material for: Comparison of clinical outcomes between sorafenib and hepatic artery infusion chemotherapy in advanced hepatocellular carcinoma: A STROBE-compliant article
Source: Medicine (Baltimore). 2018 Apr 27;97(17):e0611. doi: 10.1097/MD.0000000000010611 (PMC5944539; doi:10.1097/MD.0000000000010611)
Supplement: Supplemental Digital Content [file medi-97-e0611-s001.doc]

**Supplementary table 1.** Factors associated with the objective response in patients treated with

hepatic artery infusion chemotherapy

|  | HR | 95% CI | Univariate  (*P-*value) | Multivariate  (*P-*value) |
| --- | --- | --- | --- | --- |
| Age, years | 1.02 | 0.96-1.09 | 0.524 |  |
| Gender (male/female) | 1.28 | 0.26-4.94 | 0.731 |  |
| Platelet count, K/uL | 1.00 | 1.00-1.00 | 0.310 |  |
| Albumin, g/dL | 1.82 | 0.77-4.56 | 0.183 | .7 |
| Total bilirubin, mg/dL | 0.85 | 0.29-1.16 | 0.644 |  |
| INR | 0.35 | 0.01-3.38 | 0.453 |  |
| AFP, ng/mL | 1.50 | 0.56-4.32 | 0.436 |  |
| Largest tumor diameter, cm | 0.72 | 0.23-1.99 | 0.542 |  |
| PVT (no/yes) | 0.70 | 0.41-1.20 | 0.099 |  |
| HAIC cycles (<4/≥4) | 6.37 | 2.07-24.81 | 0.003 | .01 |

HAIC, hepatic artery infusion chemotherapy; HR: hazard ratio; CI, confidence interval; PT INR, prothrombin time international normalized ratio; AFP, alpha-fetoprotein; PVT, portal vein thrombosis.

**Supplementary table 2.** Differences in dose reduction-related adverse events between the hepatic artery infusion chemotherapy and sorafenib groups

|  | HAIC (n=95) | Sorafenib (n=44) | Total (n=139) | *P*-value |
| --- | --- | --- | --- | --- |
| Dose reduction | 44 (46.3) | 24 (54.5) | 68 (48.9) | .4 |
| Main cause of dose reduction |  |  |  |  |
| Allergic reaction | 0 (0.0) | 3 (6.8) | 3 (2.2) |  |
| Diarrhea | 1 (1.1) | 4 (9.1) | 5 (3.6) |  |
| EV hemorrhage | 5 (5.3) | 1 (2.3) | 6 (4.3) |  |
| Fatigue | 14(14.7) | 5 (11.4) | 19 (13.7) |  |
| Hand foot syndrome | 0 (0.0) | 4 (9.1) | 4 (2.9) |  |
| Hepatic failure | 9 (9.5) | 4 (9.1) | 13 (9.4) |  |
| Neutropenia | 4 (4.2) | 0 (0.0) | 4 (2.9) |  |
| Oral mucositis | 1 (1.1) | 3 (6.8) | 4 (2.9) |  |
| Pancytopenia | 3 (3.2) | 0 (0.0) | 3 (2.2) |  |
| Skin infection | 4 (4.2) | 0 (0.0) | 4 (2.9) |  |
| Thrombocytopenia | 3 (3.2) | 0 (0.0) | 3 (2.2) |  |
| Degree of dose reduction |  |  |  | .01 |
| 25% | 6 (6.3) | 0 (0.0) | 6 (4.3) |  |
| 50% | 7 (7.4) | 12 (27.3) | 19 (13.7) |  |
| Discontinuation | 31 (32.6) | 12 (27.3) | 43 (30.9) |  |

Values are presented as number (%).

HAIC, hepatic artery infusion chemotherapy; EV, esophageal varix.
